# Parity is associated with long-term differences in DNA methylation at genes related to neural plasticity in multiple sclerosis

**DOI:** 10.1186/s13148-023-01438-4

**Published:** 2023-02-10

**Authors:** Maria Pia Campagna, Alexandre Xavier, Jim Stankovich, Vicki E. Maltby, Mark Slee, Wei Z. Yeh, Trevor Kilpatrick, Rodney J. Scott, Helmut Butzkueven, Jeannette Lechner-Scott, Rodney A. Lea, Vilija G. Jokubaitis

**Affiliations:** 1 Department of Neuroscience, Monash University, Melbourne, VIC Australia; 2 School of Biomedical Sciences and Pharmacy, University of Newcastle, Newcastle, Australia; 3 Hunter Medical Research Institute, University of Newcastle, Newcastle, NSW Australia; 4 School of Medicine and Public Health, University of Newcastle, Newcastle, NSW Australia; 5 Neurology Department, John Hunter Hospital, Hunter New England, Newcastle, NSW Australia; 6 College of Medicine and Public Health, Flinders University, Adelaide, Australia; 7 Neurology Department, Alfred Health, Melbourne, VIC Australia; 8 Department of Medicine, University of Melbourne, Melbourne, VIC Australia; 9 Department of Neurology, Royal Melbourne Hospital, Melbourne, VIC Australia; 10 Queensland University of Technology, Brisbane, QLD Australia

**Keywords:** Pregnancy, Birth, Epigenetics, Neurodegeneration, Neuroplasticity

## Abstract

**Background:**

Pregnancy in women with multiple sclerosis (wwMS) is associated with a reduction of long-term disability progression. The mechanism that drives this effect is unknown, but converging evidence suggests a role for epigenetic mechanisms altering immune and/or central nervous system function. In this study, we aimed to identify whole blood and immune cell-specific DNA methylation patterns associated with parity in relapse-onset MS.

**Results:**

We investigated the association between whole blood and immune cell-type-specific genome-wide methylation patterns and parity in 192 women with relapse-onset MS, matched for age and disease severity. The median time from last pregnancy to blood collection was 16.7 years (range = 1.5–44.4 years). We identified 2965 differentially methylated positions in whole blood, 68.5% of which were hypermethylated in parous women; together with two differentially methylated regions on Chromosomes 17 and 19 which mapped to TMC8 and ZNF577, respectively. Our findings validated 22 DMPs and 366 differentially methylated genes from existing literature on epigenetic changes associated with parity in wwMS. Differentially methylated genes in whole blood were enriched in neuronal structure and growth-related pathways. Immune cell-type-specific analysis using cell-type proportion estimates from statistical deconvolution of whole blood revealed further differential methylation in T cells specifically (four in CD4^+^ and eight in CD8^+^ T cells). We further identified reduced methylation age acceleration in parous women, demonstrating slower biological aging compared to nulligravida women.

**Conclusion:**

Differential methylation at genes related to neural plasticity offers a potential molecular mechanism driving the long-term effect of pregnancy on MS outcomes. Our results point to a potential ‘CNS signature’ of methylation in peripheral immune cells, as previously described in relation to MS progression, induced by parity. As the first epigenome-wide association study of parity in wwMS reported, validation studies are needed to confirm our findings.

**Supplementary Information:**

The online version contains supplementary material available at 10.1186/s13148-023-01438-4.

## Introduction

Multiple sclerosis (MS) is most prevalent in females, with a sex ratio of 3:1 [1]. It is frequently diagnosed between 20 and 40 years of age, the prime reproductive years for women. Understanding the effect of pregnancy on disease activity and progression is a priority for women with MS (wwMS) and their care teams.

Changes to MS disease activity during pregnancy and the post-partum are well understood. Relapse rates decrease throughout pregnancy and are lowest in the third trimester. This decrease is more pronounced for women with mild disease than those with severe disease who discontinue moderately to highly effective disease-modifying therapies (DMTs) during pregnancy [2]. A subsequent increase in post-partum relapse rate is observed, with approximately 14% of women relapsing in the modern DMT era [2]. Evidence shows that pregnancy-related changes in disease activity are driven by the immunomodulatory effects of pregnancy hormones, particularly estriol (E3) which is present in 300-fold concentration compared to non-pregnancy [3]. Pregnancy is not detrimental to long-term MS outcomes [4–17], with some studies demonstrating a pregnancy benefit [18–24]. One of the largest real-world studies in 1830 wwMS found that one or more pregnancies after disease onset were associated with a modest, but significant, 0.36 point lower Expanded Disability Status Scale (EDSS) score compared to nulligravida women over a ten-year follow-up [18]. Notably, the protective effect of pregnancy in this cohort was fourfold greater than that of first-line DMT exposure in the same timeframe [18]. Furthermore, a study of 2557 wwMS showed that a history of childbirth delayed the onset of a clinically isolated syndrome (CIS, the first demyelinating event indicative of a future MS diagnosis) by 3.4 years [25]. Unlike intrapartum disease activity, the biological mechanisms underpinning these long-term effects of pregnancy are not understood. As these effects have been shown to last years beyond birth [18], they cannot be explained by pregnancy-related hormonal changes exclusively which have been shown to return to pre-pregnancy levels by six months post-partum [26].

Epigenetic mechanisms regulate gene expression in a dynamic and reversible manner. DNA methylation (DNAm) is a key epigenetic mechanism. The absence or presence of a methyl group on cytosine-phosphate-guanine (CpG) dinucleotides generally activates or represses gene transcription, respectively. Epigenetic mechanisms are influenced by life events and environmental factors, including the multitude of physiological and hormonal changes of pregnancy. DNA methylation enzymes are specifically influenced by oestrogen signalling, which increases in pregnancy and peaks in the third trimester. Converging evidence outlines a role for DNA methylation in the effect of pregnancy on outcomes in wwMS through altering immune and central nervous system (CNS) function: (1) oestrogen signalling influences DNA methylation enzymes [27], (2) pregnancy has been shown to reduce immune epigenetic age in women without MS [28], and (3) pregnancy induces changes in the expression of immune-activation [29] and axon-guidance [30] genes in wwMS for up to 19 years after pregnancy. However, no epigenome-wide association study (EWAS) of parity in wwMS has been reported to date.

The objective of this study was to understand the long-term impact of parity on DNA methylation patterns in women with relapse-onset MS. We first sought to identify whole blood and immune cell-specific DNA methylation patterns, across autosomes, associated with parity. Secondly, we aimed to compare methylation age acceleration (MAA) between nulligravida and parous wwMS, to determine whether reductions in MAA reported in health were also evident in an MS cohort.

## Results

### Cohort descriptive statistics

We included 192 women with relapse-onset MS (RMS) across four study sites (nulligravida = 96, parous = 96, Additional file 1: Fig. S1). Participants were categorised based on available pregnancy history data from the MSBase Registry [31, 32] and matched by age, geographical location and disease severity. We excluded women who had a known history of pregnancy ending in miscarriage or termination. Therefore, the parous group included women with term or preterm births, and the nulligravida group included women who had never been pregnant. The median time from last conception to blood collection in the parous group was 16.66 years (range = 1.45–44.42 years, Table 1).

**Table 1:** Cohort summary statistics

Characteristics	Nulligravida (*n* = 96)	Parous (*n* = 96)	All (*n* = 192)	Cohen’s *d*
Time from last pregnancy to blood collection (years)				
Median (IQR)	NA	16.66 (9.13, 27.66)	NA	–
Range	NA	1.45–44.42	NA	
ARMSS score				
Median (IQR)	6.63 (1.47, 8.73)	7.08 (1.29, 8.22)	6.99 (1.39, 8.37)	0.01
Range	0.16–9.55	0.19–9.91	0.16–9.91	
Disease course				
RRMS	57 (60.0%)	63 (66.3%)	120 (63.2%)	NA
SPMS	38 (40.0%)	32 (33.7%)	70 (36.8%)	
Sex				
Female	124 (100.0%)	96 (100.0%)	220 (100.0%)	NA
Male	0 (0%)	0 (0%)	0 (0%)	
Age at most recent visit				
Median (IQR)	48.3 (40.7, 56.6)	48.6 (39.5, 57.2)	48.9 (40.7, 57.1)	0.03
Range	27.6–70.6	24.2–69.8	24.2–70.6	
Age at blood collection				
Median (IQR)	48.7 (41.2, 57.0)	48.9 (40.3, 57.9)	48.9 (40.7, 57.1)	0.03
Range	28.3–70.6	26.8–69.8	26.8–70.6	
Follow-up in MSBase (years)				
Median (IQR)	6.26 (3.46, 8.91)	6.92 (5.51, 9.42)	6.54 (4.16, 8.99)	0.26
Range	0.00–24.80	0.00–19.30	0.00–24.80	
Number of EDSS scores assessed				
Median (IQR)	7.5 (4.0, 9.0)	8.5 (6.0, 9.0)	8.0 (5.0, 9.0)	0.35
Range	1.0–9.0	1.0–9.0	1.0–9.0	
Symptom duration (years)				
Median (IQR)	15.77 (9.62, 24.60)	14.98 (8.84, 20.65)	15.11 (9.26, 22.15)	0.15
Range	1.01–42.37	0.21–41.56	0.21–42.37	
ARR in preceding year				
Median (IQR)	0.0 (0.0–0.0)	0.0 (0.0–0.0)	0.0 (0.0–0.0)	0.08
Range	0.0–3.0	0.0–2.0	0.0–3.0	
Number of births				
0	96 (100%)	0 (0%)	96 (50%)	–
1	0 (0%)	29 (30%)	29 (15%)	
2	0 (0%)	46 (48%)	46 (24%)	
3	0 (0%)	14 (15%)	14 (7%)	
≥ 4	0 (0%)	7 (7%)	7 (4%)	
DMT				
None	37 (38%)	34 (35%)	71 (37%)	–
Alemtuzumab	0 (0%)	1 (1%)	1 (1%)	
Dimethyl fumarate	2 (2%)	8 (8%)	10 (5%)	
Fingolimod	21 (22%)	25 (26%)	46 (24%)	
Glatiramer acetate	7 (7%)	3 (3%)	10 (5%)	
Interferon beta	12 (13%)	10 (11%)	22 (11%)	
Natalizumab	17 (18%)	11 (12%)	28 (15%)	
Teriflunomide	0 (0%)	4 (4%)	4 (2%)	
Smoking history				
Ever	16 (16.7%)	39 (40.6%)	55 (28.6%)	–
Never	17 (17.7%)	28 (29.2%)	45 (23.4%)	
Unknown	63 (65.6%)	29 (30.2%)	92 (47.9%)	
DNAmPACKYRS^a^				
Median (IQR)	0.93 (− 3.9, 8.5)	0.60 (− 4.6, 13.6)	0.85 (− 4.3, 9.8)	0.20
Range	− 12.6–33.7	− 13.5–32.6	− 13.5–33.7	
CD4^+^ cell proportions^b^				
Median (IQR)	0.07 (0.05, 0.11)	0.08 (0.05, 0.11)	0.08 (0.05, 0.11)	0.06
Range	0.01–0.23	0.00–0.26	0.0–0.26	
CD8^+^ cell proportions^b^				
Median (IQR)	0.03 (0.01–0.05)	0.03 (0.01, 0.05)	0.03 (0.09, 0.13)	0.13
Range	0.00–0.10	0.00–0.18	0.04–0.25	
NK cell proportions^b^				
Median (IQR)	0.08 (0.06, 0.09)	0.08 (0.06, 0.10)	0.08 (0.06, 0.10)	0.41
Range	0.02–0.13	0.04–0.21	0.02–0.21	
B cell proportions^b^				
Median (IQR)	0.10 (0.08, 0.11)	0.09 (0.08, 0.12)	0.08 (0.06, 0.10)	0.09
Range	0.03–0.16	0.05–0.16	0.02–0.21	
Monocyte proportions^b^				
Median (IQR)	0.10 (0.09, 0.13)	0.11 (0.09, 0.13)	0.11 (0.09, 0.13)	0.17
Range	0.04–0.21	0.04–0.25	0.04–0.25	
Granulocyte proportions^b^				
Median (IQR)	0.60 (0.40, 0.67)	0.60 (0.54, 0.66)	0.60 (0.54, 0.66)	0.14
Range	0.40–0.79	0.31–0.78	0.31–0.79	

ARMSS, Age-Related Multiple Sclerosis Severity Score; IQR, interquartile range; EDSS, Expanded Disability Status Scale; ARR, annualised relapse rate; DMT, disease-modifying therapy; DNAmPACKYRS, DNA methylation estimate of smoking pack years; and NK, natural killer

^a^A DNA methylation biomarker of smoking estimated using methylation levels at smoking-associated CpGs. Calculated online at https://dnamage.genetics.ucla.edu/home

^b^Estimated using reference-based statistical deconvolution with the EpiDISH R package and CIBERSORT algorithm

### Differential methylation analysis—whole blood

After methylation data pre-processing, approximately 747,000 (86%) of 867,000 probes remained for differential methylation analysis (Additional file 1: Fig. S2a–d). Batch effect analysis identified Plate, Sentrix ID and Sentrix Position as significant sources of technical variation (p < 0.01), which were corrected and reduced to negligible effects using the *Combat* algorithm [33] (Additional file 1: Fig. S2e–h).

We conducted a whole blood EWAS adjusted for immune cell-type proportions (CD4^+^ T cells, CD8^+^ T cells, B cells, natural killer (NK) cells, granulocytes and monocytes) and DNAmPACKYRS due to non-negligible differences between groups (Table 1). DNAmPACKYRS is an accurate biomarker of smoking history based on methylation at a subset of smoking-associated CpGs [34]. This analysis identified 2965 differentially methylated positions (DMPs) surpassing genome-wide thresholds (FDR < 0.05 and Δ_meth_ > 1%), and Table 2 shows the top 10 DMPs by effect size. (Full list is available in Additional file 2: Table S1.) Of 2965 DMPs, 1395 mapped to genes and 1570 to intergenic regions or unannotated locations. Moreover, 2046 (68.5%) DMPs were hypermethylated and 940 (31.5%) were hypomethylated in the parous group, relative to the nulligravida group (Additional file 1: Fig. S3). Methylation beta values equate to percentage methylation. Therefore, we report methylation differences (effect size, Δ_meth_) as a percentage going forward (e.g. Δ_meth_ of 0.01 = 1%). Δ_meth_ ranged from − 16.1 to 14.0%. Methylation is most likely to impact gene transcription when located in CpG islands. Only 326 (10.9%) of DMPs mapped to CpG islands, with 608 (20.4%) in shores, 223 (7.5%) in shelves and the majority in open sea regions (1829, 61.3%). Compared to the locations of the 746,969 tested CpGs (islands = 143,779 (19.2%), shores = 136,076 (18.2%), shelves = 51,041 (6.8%), open sea regions = 416,073 (55.7%)), our DMPs show slight overrepresentation in open sea regions and underrepresentation in islands. We validated 32 DMPs from Mehta et al. [30] (Table 3), 22 with the same direction of effect (DOE), and 366 genes (Additional file 2: Table S2), 104 (28.4%) with the same DOE (Additional file 2: Table S3).

**Table 2:** Top 10 differentially methylated positions (DMPs) ranked by absolute effect size (∆_meth_)

CpG	Chr	Position (bp)	Gene	Feature	∆_meth*_	FDR
cg12036633	15	63758958		IGR	− 0.161	0.032
cg27484945	19	15561759		TSS1500	− 0.153	0.042
cg08779649	13	50194554		IGR	0.140	0.043
cg02122327	13	50194322		IGR	0.133	0.015
cg21415084	12	84218134	EPB41L5	IGR	0.114	0.039
cg03885684	2	120770471		TSS200	0.108	0.006
cg24000535	14	91110600	PRKCE	Body	− 0.083	0.017
cg08166072	2	46213920		Body	0.080	0.012
cg14248704	5	151470842		Body	0.074	0.006
cg10140164	9	75597328	IGR		− 0.072	0.017

CpG, cytosine-phosphate-guanine; Chr, chromosome; bp, base pair; IGR, intergenic region; TSS200, transcript start site (up to 200 bp 5′ of 5′UTR) promoter region; and TSS1500, transcript start site (up to 1500 bp 5′ of 5′UTR) promoter region

^*^Nulligravida to parous

**Table 3:** Validated CpGs from Mehta et al. [30] ordered by chromosomal position

CpG	Chr	Position (bp)	Gene	This study		Mehta et al. [30]		DOE validation
				∆_meth*_	FDR	∆_meth*_	*p* value	
cg15132013	1	2163529	SKI	0.028	0.042	− 0.083	0.043	No
cg00935967	1	3822762	LOC100133612	0.018	0.048	− 0.047	0.028	No
cg12385729	1	9243679		− 0.025	0.038	0.140	0.046	No
cg22332037	1	153951279	JTB	− 0.013	0.025	0.028	0.02	No
cg17403266	1	226719677		0.011	0.037	− 0.027	0.046	No
cg20848488	4	6417932	PPP2R2C	0.025	0.026	− 0.115	0.018	No
cg05104897	4	120127485		0.029	0.012	− 0.097	0.031	No
cg19083007	6	46293862	RCAN2	0.016	0.027	− 0.049	0.026	No
cg22390040	8	17220621	MTMR7	0.017	0.049	− 0.062	0.012	No
cg03460027	10	390328	DIP2C	0.01	0.026	− 0.040	0.003	No
cg03004330	10	13934438	FRMD4A	− 0.017	0.041	0.180	0.024	No
cg21937554	11	44331934	ALX4	− 0.024	0.045	0.305	0.043	No
cg11933951	11	82997796	CCDC90B	0.022	0.028	− 0.062	3.78 × 10^−4^	No
cg16984132	11	117052181	SIDT2	− 0.011	0.025	− 0.103	0.031	Hypo
cg23031939	11	134216076	GLB1L2	− 0.014	0.044	− 0.179	0.005	Hypo
cg10812526	12	101192122	ANO4	0.037	0.05	0.056	0.041	Hyper
cg01903420	13	27295928		0.049	0.043	− 0.397	0.048	No
cg08419873	13	27296010		0.072	0.031	− 0.386	0.049	No
cg15288350	13	113987359	GRTP1	− 0.011	0.037	− 0.033	0.038	Hypo
cg26647324	16	1516388	CLCN7	0.011	0.046	− 0.023	0.031	No
cg00356208	16	55167581		0.016	0.016	− 0.082	0.034	No
cg09663193	17	7486821	MPDU1	− 0.016	0.031	− 0.124	0.029	Hypo
cg14121185	17	64488849	PRKCA	0.026	0.037	− 0.079	0.032	No
cg05229454	17	80494379	FOXK2	0.031	0.022	− 0.202	0.010	No
cg23973972	18	72152075		0.034	0.008	− 0.090	0.039	No
cg08134671	19	2542837	GNG7	0.035	0.015	− 0.104	0.038	No
cg10669058	19	19648555	CILP2	− 0.029	0.048	− 0.171	0.022	Hypo
cg14166009	19	37825309	HKR1	− 0.022	0.042	− 0.100	0.044	Hypo
cg13687570	19	37825320	HKR1	− 0.028	0.044	− 0.170	0.008	Hypo
cg04442417	20	62191507	HELZ2	− 0.033	0.003	− 0.073	0.025	Hypo
cg02836046	21	38120824	SIM2	− 0.024	0.022	− 0.059	0.044	Hypo
cg11314536	22	18033434	CECR2	0.017	0.034	− 0.046	0.010	No

CpG, cytosine-phosphate-guanine; Chr, chromosome; bp, base pair; FDR, false discovery rate; and DOE, direction of effect

^*^Nulligravida to parous

No differentially methylated regions (DMRs) were identified using the *DMRcate* algorithm at an FDR threshold of 0.05. Therefore, we identified DMRs from our DMP list, defining a DMR as a region containing at least five DMPs with the same effect direction and FDR < 0.01, within 1000 bp of the adjacent DMPs [35]. Using this definition, we identified two DMRs on Chromosomes 17 and 19 (Table 4, Additional file 1: Fig. S4). DMR^Chr^ [17] mapped to TMC8 and showed hypermethylation in the parous group. DMR^Chr^ [19] mapped to ZNF577 and was hypomethylated in the parous group.

**Table 4:** Differentially methylated regions (DMRs)

Chr	Region (bp)	Width (bp)	Gene	Feature	CpGs	∆_max_	∆_mean_
17	76128906–76130139	1233	TMC8	Body/TSS1500	cg16301617 cg16935597 cg14055168 cg04945945 cg22833809 cg01791634 cg18437480	0.017	0.013
19	52390810–52391789	979	ZNF577	Body/TSS200/TSS1500	cg06878361 cg03562414 cg10635122 cg24794228 cg10783469 cg16731240 cg23010048 cg11269599 cg22331349 cg09547119 cg12227172 cg22472290 cg13393830 cg25361850	− 0.033	− 0.023

Chr, chromosome; bp, base pair; DMP, differentially methylated position; CpG, cytosine-phosphate-guanine; max, maximum; TMC8, transmembrane channel like 8; ZNF577, zinc finger protein 577; and TSS1500, transcript start site (up to 1500 bp 5′ of 5′UTR) promoter region

### Differential methylation analysis—immune cell specific

DNA methylation can be cell-type specific. Therefore, whole blood analysis can be (1) confounded by cell-type proportions or (2) insensitive to cell-specific DMPs associated with the outcome. To address these limitations of whole blood analysis, we estimated and compared the proportion of immune cell types between groups using the reference-based *CIBERSORT* algorithm [36]. There were differences in the proportions of CD8^+^ T cells (Cohen’s *d* = 0.13), NK cells (Cohen’s *d* = 0.41) and monocytes (Cohen’s *d* = 0.17, Table 1). To address limitation one, we adjusted the whole blood analysis for all cell-type proportions. To address the second limitation, we used the cell-type proportion estimates to identify cell-type-specific DMPs (csDMPs) using a separate linear model for each cell type where the outcome was methylation M-value, and the predictors were cell-type proportion estimate and an interaction term of cell-type proportion and parity. This revealed four CD4^+^ and eight CD8^+^ T cell-specific DMPs (Table 5). All CD4^+^ T cell DMPs were hypermethylated in the parous group compared to the nulligravida group, with only one DMP mapping to a gene (cg14172633, *HMCN1*). In CD8^+^ T cells, three DMPs were hypermethylated and five were hypomethylated in the parous group. DMP cg25577322 had the largest effect size (estimate = -8.32, SE = 1.45) and mapped to *AHR*. Seven of the eight DMPs mapped to a gene, and two DMPs mapped to *OR2L13* (cg08944170 and cg20507276).

**Table 5:** Cell-specific differentially methylated positions (csDMPs)

CpG	Chr	Position (bp)	Gene	Feature	Nulligravida mean*	Parous mean*	Est	SE	p
*CD4*^*+*^* T cells*									
cg14172633	1	185703557	*HMCN1*	TSS200	− 3.10	− 2.84	− 3.64	0.62	1.7 × 10^−8^
cg15145296	3	125709740			− 4.24	− 3.98	− 3.23	0.56	3.6 × 10^−8^
cg06032337	6	29648468			− 2.82	− 2.71	− 3.17	0.56	5.1 × 10^−8^
cg06818823	6	46459236			− 6.55	− 5.90	− 5.88	1.04	5.8 × 10^−8^
*CD8*^*+*^* T cells*									
cg01858500	17	68202566			− 3.57	− 3.52	− 4.27	0.71	1.1 × 10^−8^
cg08944170	1	248100614	*OR2L13*	1stExon	− 3.38	− 3.60	− 2.78	0.48	2.3 × 10^−8^
cg25577322	7	17338213	*AHR*	TSS200	− 6.33	− 6.94	− 8.32	1.45	4.0 × 10^−8^
cg16402757	10	35311004	*CUL2*	Body	− 2.14	− 2.05	− 2.14	0.38	4.6 × 10^−8^
cg03495768	13	100637113	*ZIC2*	Body	− 2.98	− 3.07	− 3.05	0.54	6.1 × 10^−8^
cg04798314	1	246668601	*SMYD3*	Body	− 2.35	− 2.56	− 2.71	0.48	6.7 × 10^−8^
cg11738485	19	12877000	*HOOK2*	Body	− 2.79	− 2.51	− 2.42	0.43	6.8 × 10^−8^
cg20507276	1	248100600	*OR2L13*	1stExon	− 3.40	− 3.55	− 2.68	0.48	8.6 × 10^−8^

CpG, cytosine-phosphate-guanine; Chr, chromosome; bp, base pair; Est, estimate (from linear regression); SE, standard error; and TSS200, transcript start site (up to 200 bp 5′ of 5′UTR) promoter region

^*^Mean M-values reported as M-values used in cell-specific statistical analyses

### Sensitivity analysis

We performed sensitivity analyses to assess the potential impact of demographic, clinical, biological and environmental covariates on the primary methylation analysis by testing the association between covariates and genome-wide methylation. Genome-wide methylation was not associated with symptom duration, annualised relapse rate (ARR), methylation age acceleration (PhenoAge and GrimAge) or DMT at blood collection (yes or no). Consequently, these covariates were not included in the differential methylation analyses so as not to unnecessarily burden the model and reduce statistical power.

### Targeted methylation quantitative trait loci (mQTL) analyses

Differential methylation between sample groups can be driven by mQTLs—loci at which the underlying single nucleotide variants (SNVs) at or near that site significantly influence methylation levels at a nearby CpG. If mQTLs are not accounted for, differential methylation signals may actually be underlying differences in genotype between sample groups. Therefore, we tested the relationship between genotype and methylation at CpGs within a ± 5 kb window of each DMR to determine if differential methylation was associated with, or independent of, underlying genotype.

After quality control and filtering, 183 patients had available genotype data and remained for analysis. DMR^Chr^ [17] contained 13 SNVs, three of which were independent based on linkage disequilibrium (LD) analysis (Additional file 2: Table S4a). Methylation at all DMR CpGs except cg16935597 was associated with genotype at two or more of the three independent SNVs (Kruskal test p values in Additional file 2: Table S4b). After accounting for genotype at these mQTLs, methylation at all CpGs within DMR^Chr^ [17] remained associated with parity (general linear model p values in Additional file 2: Table S4b).

DMR^Chr^ [19] contained 10 SNVs, four of which were independent based on linkage disequilibrium (LD) analysis (Additional file 2: Table S5a). Methylation at all DMR CpGs, except cg06878361, cg10635122 and cg16731240, was associated with genotype at all four SNVs (Kruskal test p values in Additional file 2: Table S5b). After accounting for genotype at these mQTLs, methylation at all CpGs within DMR^Chr^ [19] remained associated with parity (general linear model p values in Additional file 2: Table S5b).

### Multi-factor feature selection

Elastic net regression is a form of penalised regression that is useful for uncovering multiple conjoint effects in datasets with correlated features (e.g. methylation) and a greater number of features than samples (*p* >>> *n*). This method can be useful for identifying important features with greater sensitivity than conventional EWAS analyses. Using elastic net regression, we identified a panel of CpGs conferring a conjoint association. We determined the optimal alpha (0.1) and lambda (0.02) values for our data using a cross validation approach. With an alpha value closer to zero than one, our elastic net regression resembled a lasso regression more closely than a ridge regression.

Using these model parameters, our elastic net regression model selected 1505 CpGs associated with parity (top 10 shown in Table 6, full list in Additional file 2: Table S6) in our training dataset (*n* = 134, 70% of cohort). Of these, 316 CpGs were also identified as DMPs in our primary analysis.

**Table 6:** Top ten CpGs associated with parity as selected by the elastic net model based on variable importance

CpG	Chr	Position (bp)	Gene	Feature	Importance
cg26506013	16	28887830		IGR	100
cg25485991	17	8066461	VAMP2	TSS200	99.92
cg23367339	17	36622717	ARHGAP23	Body	99.91
cg08186508	14	68067006	PIGH	5′UTR	99.84
cg17070338	13	111268441	CARKD	Body	99.82
cg07360021	6	151186904	MTHFD1L	1stExon	99.68
cg23841819	1	204970383	NFASC	Body	99.48
cg11918372	2	48132755	FBXO11	5′UTR	99.24
cg27573735	3	82857144			99.08
cg12835012	4	183795785			98.91

Chr, Chromosome; bp, base pair; IGR, intergenic region; TSS200, transcript start site (up to 200 bp 5′ of 5′UTR), promoter region; and 5′UTR, 5′ untranslated region

### Gene set enrichment analysis (GSEA)

We conducted GSEA using (a) the 104 genes validated from Mehta et al. [30] with the same direction of effect (Additional file 2: Table S3) and (b) all 1395 genes identified in the primary whole blood analysis and by multifactor feature selection to elucidate potentially small but cumulative effects of parity on methylation patterns (Additional file 2: Table S7).

**Fig. 1:**
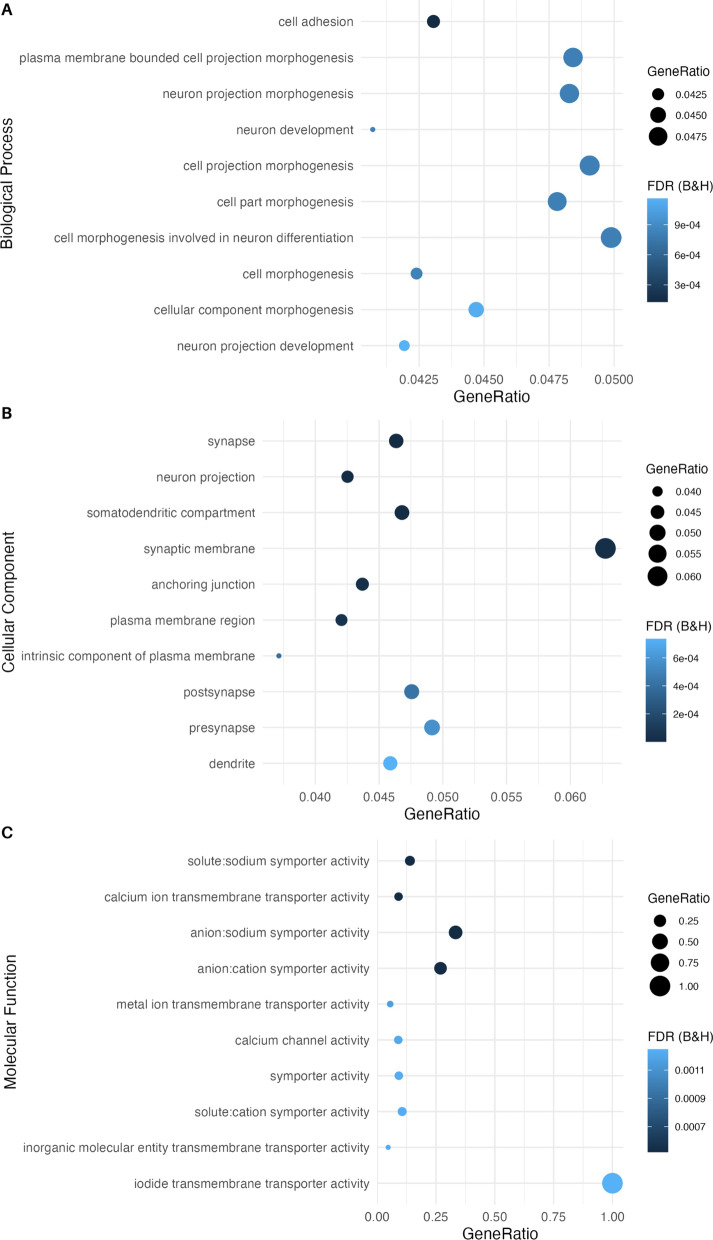
Gene set enrichment analysis of overlapping differentially methylated positions from the differential methylation and elastic net analyses. Input data were genes identified in both the differential methylation analysis and elastic net regression (*n* = 2103). The **A** ten most significantly enriched biological processes, **B** ten most significantly enriched cellular compartments, **C** ten most significantly enriched molecular functions. Gene ratio is the ratio of the number of genes in the query list and the hit count for that gene set in the genome

Validated genes from Mehta et al. [30] (*n* = 366) were enriched in synapse cellular components (Additional file 1: Fig. S5a) and embryogenesis biological processes (Additional file 1: Fig. S5b), including neuron projection (*n*_genes_ = 53, FDR_B&H_ = 1.07 × 10^−3^) and central nervous system development (*n*_genes_ = 53, FDR_B&H_ = 0.005). Using genes identified in the primary whole blood analysis and by multifactor feature selection (*n* = 2103), we revealed that differential methylation, regardless of direction of effect, was primarily enriched in biological processes (Fig. 1a) and cellular compartments (Fig. 1b) related to neuronal growth. This included neuron projection morphogenesis (*n*_genes_ = 45, FDR_B&H_ = 0.0008), neuron development (*n*_genes_ = 68, FDR_B&H_ = 0.0008) and neuron projection (*n*_genes_ = 76, FDR_B&H_ = 4.5 × 10^−6^). Furthermore, the top enriched molecular functions related to ion transport including anion/cation symporter activity (*n*_genes_ = 7, FDR_B&H_ = 0.0005) and calcium channel activity (*n*_genes_ = 15, FDR_B&H_ = 0.001, Fig. 1c). There were no enriched gene ontology terms using GOmeth with an FDR threshold of 0.05. This suggests that our ToppGene findings could be a result of probe number or multi-gene bias. However, we used GSEA as an exploratory analysis to generate hypotheses about the mechanism in which pregnancy impacts clinical outcomes and have therefore interpreted the results with caution.

### Methylation age analysis

Methylation age acceleration (MAA) measures the disparity between chronological and biological age as estimated using methylation age algorithms and can provide insight into an individual’s health and lifespan [34, 37, 38]. As groups were a priori matched by age, there were no significant differences in chronological age between groups (Table 1). The correlation between chronological age and methylation age using the PhenoAge and GrimAge algorithms were 0.77 and 0.91, respectively. We did not find any evidence for differences in methylation age between groups using the GrimAge algorithm (*p* = 0.854). However, we did find significant differences in methylation age between groups using the PhenoAge algorithm (*p* = 0.034, Additional file 1: Fig. S6).

**Fig. 2:**
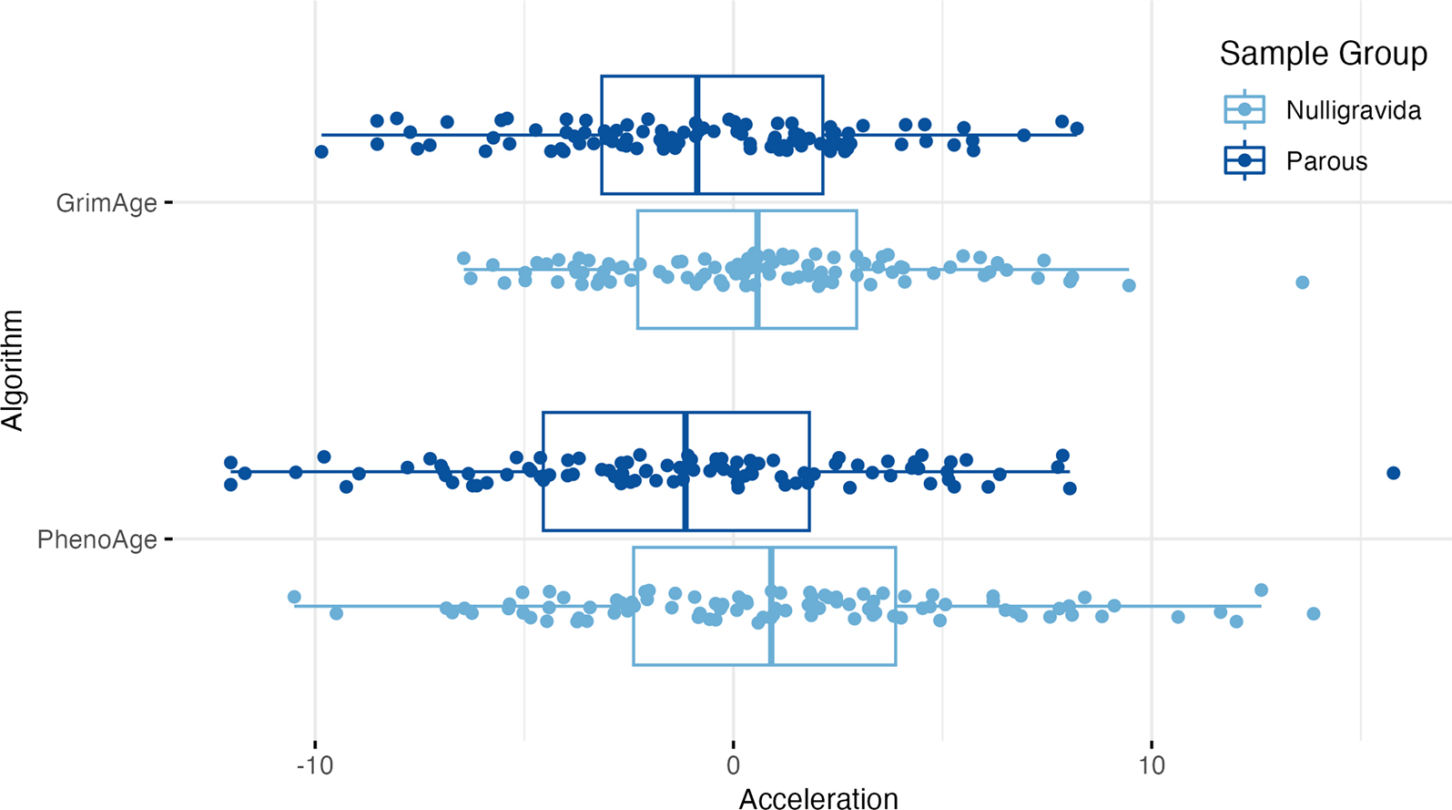
PhenoAge and GrimAge acceleration by sample group. There are significant differences in methylation age acceleration between nulligravida and parous groups using both the PhenoAge (Δ*μ* = 2.27 years, standard error (SE) = 0.50, *p* = 0.001) and GrimAge (Δ*μ* = 1.44 years, SE = 0.80, *p* = 0. 0.005) algorithms

MAA was calculated as the residual term from regressing chronological age on methylation age. Residual terms were normally distributed for the PhenoAge (*p* = 0.551) algorithm, but not the GrimAge algorithm (*p* = 3.52 × 10^−05^). There were significant differences in MAA between nulligravida and parous groups using both the PhenoAge (Δμ = 2.27 years, *p* = 0.001) and GrimAge algorithms (Δμ = 1.44 years, *p* = 0.005, Fig. 2). There was no association between GrimAge or PhenoAge MAA and years since conception (GrimAge: *r* = 0.046, *p* = 0.654; PhenoAge: *r* = 0.057, *p* = 0.578), or menopause (*n* < 50y.o. = 92, *n* ≥ 50y.o. = 100, GrimAge: *p* = 0.574, PhenoAge: *p* = 0.714).

## Discussion

Studies have demonstrated an association between pregnancy and reduced disability accumulation in women with MS (wwMS) [39], lasting for up to ten years post-pregnancy [18]. Recent studies have identified associations between birth history and methylation patterns in health [40–42] and MS [30], as well as negative associations between birth history and methylation age acceleration in women without MS [28]. No epigenome-wide studies to date have examined parity, or methylation age acceleration in wwMS.

Our primary EWAS of whole blood methylation differences between nulligravida and parous wwMS identified 2965 differentially methylated positions (DMPs). Of these, 32 overlapped with those previously identified by Mehta et al. [30] at the CpG level, and 366 at the gene level. One CpG was hypermethylated across both studies and mapped to *ANO4*, encoding a chloride channel involved in ion channel transport. Upregulated *ANO4* expression in chronically active MS lesions compared to inactive lesions has been shown [43]. *ANO4* hypermethylation and potential downregulation in parous wwMS in this study may indicate less neuroinflammation and associated disability. Nevertheless, gene transcription and expression remain to be studied in this cohort. Validated, hypomethylated CpGs mapped to *SIDT2, GLB1L2, GRTP1, MPDU1, CILP2, HELZ2* and *SIM2*. Of these, *SIM2* is the most relevant as a transcription factor and master regulator of central nervous system development and neurogenesis [44]. A link between neurogenesis, neuronal reserve and MS outcomes has been previously suggested [45], with recent corroboration [46]. In addition to the above-mentioned genes, two validated, hypomethylated CpGs mapped to *HKR1* (alias *ZNF875*), a protein coding gene involved in transcriptional regulation and shown to be associated with maternal smoking [47], age [48] and Alzheimer’s disease (AD) pathology [49]. A 2011 study of 36 women found an association between maternal smoking and *HKR1* hypomethylation in the placenta [47], although to date, this finding has not been validated in peripheral blood. Given the lack of evidence in peripheral blood, and the adjustment of our differential methylation analysis for maternal smoking using a DNA methylation biomarker (DNAmPACKYRS), HKR1 is likely a genuine association with parity rather than a confounded result driven by smoking history. Interestingly, a 2018 study of 12 centenarians demonstrated a strong association between age in centenarians and *HKR1* hypermethylation [48]. Here, we matched nulligravida and parous groups within three years of age due to the known associations between age and methylation patterns, eliminating age as a confounding variable and potential driver of the association with *HKR1* methylation. Lastly, a 2019 study of post-mortem brain tissue from 26 AD patients showed strong associations between *HKR1* hypermethylation and AD-related pathological changes in the hippocampus. This association between neurodegeneration and *HKR1* hypermethylation begs the question of whether our findings of *HKR1* hypomethylation in peripheral blood correlate with less degeneration in CNS tissue of parous wwMS. While highly speculative, this finding brings forth an interesting avenue for further neuroimaging-based research.

Exploratory gene set enrichment analysis (GSEA) of validated genes with the same direction of effect between this study and Mehta et al. [30] highlighted synapse and extracellular matrix (ECM)-related cellular components and embryogenesis-related biological processes. ECM changes are associated with both MS-related neuroinflammation and neurodegeneration [50], as well as pregnancy [51]. The enrichment of neuronal cellular compartments alongside ECM cellular compartments suggests that validated differentially methylated genes between nulligravida and parous wwMS are enriched in genes relevant to MS-related neuroinflammation and neurodegeneration. Interestingly, the enrichment of embryogenesis-related biological processes demonstrates that methylation changes required for successful foetal development are lasting in the maternal methylome. Differences in cohort size and study design between this study and Mehta et al. [30] must be highlighted. Mehta et al. [30] sought to identify DMPs in genes that were identified a priori after gene expression analyses [30], compared to our genome-wide approach. Furthermore, they included women with a history of pregnancy, compared to our study which included only women with a history of birth. We further conducted GSEA on the 2103 differentially methylated genes identified in the primary analysis and elastic net regression. Hypomethylated genes in parous wwMS were enriched in neuron development and growth biological processes and cellular compartments, while hypermethylated genes were enriched in signal transduction biological processes and molecular functions. Mehta et al. [30] similarly found enrichment of neuronal pathways including axon guidance in their study of differentially expressed genes between nulliparous and parous wwMS [30]. While the majority of DMPs in our primary differential methylation analysis had small effect sizes, the strength of our penalised regression approach is the ability to reveal small, correlated relationships between features. Taken together, these findings suggest that methylation impacts neuro-axonal maintenance and neurite growth in parous women in a small but cumulative manner, up to 44.4 years after pregnancy. These findings are consistent with reports that the brains of women who have children undergo pronounced morphological changes as a result of pregnancy [52]. Moreover, single nucleotide variants associated with CNS function were recently shown to associate with MS severity outcomes [53]. Therefore, our study confirms that genes related to neuronal processes are differentially methylated between nulligravida and parous wwMS and demonstrates a putative mechanism by which pregnancy may impact long-term legacy effects on outcomes in wwMS.

In addition to 2965 DMPs, we identified two differentially methylated regions (DMRs) on Chromosomes 17 and 19. DMR^Chr^ [17] contains seven hypermethylated DMPs in the gene body and transcript start site (up to 1500 bp 5′ of 5′UTR) promoter region of *TMC8,* a protein coding gene thought to be involved in CD4^+^ T cell regulation due to its role in regulating cervical cancer susceptibility [54] and head and neck squamous cell cancer prognosis [55]. A role for this gene in MS or pregnancy has not been established. DMR^Chr^ [19] contains 14 hypomethylated DMPs in the gene body and transcript start site (up to 1500 bp 5′ of 5′UTR) promoter region of *ZNF577*, a zinc finger protein coding gene involved transcriptional regulation. *ZNF577* is a breast cancer risk gene in European populations [56], at which hypermethylation is associated with obesity and post-menopausal status in breast cancer tissue [57] and leukocytes [58]. Due to age-matching our nulligravida and parous groups, we are confident that this finding is not driven by menopause status; however, we were unable to test the effect of obesity due to lack of data. Our findings require validation in an independent cohort where replicated DMR signals would provide a rationale for in vitro functional studies of gene and protein expression control mediated by each DMR.

Using reference-based statistical deconvolution of whole blood methylation data, we identified four CD4^+^ T cell-specific DMPs (csDMPs). CD4^+^ T cells are central to immune regulation and tolerance and have been strongly linked to both MS [59] and pregnancy [60]. Multiple studies have reported changes in the epigenetic patterns of CD4^+^ T cells during pregnancy in wwMS [29, 61, 62]. The only gene-associated DMP was at the transcription start site for *HMCN1*, a member of the immunoglobulin superfamily. To date, *HMCN1* has not been associated with differential methylation in healthy populations or wwMS during pregnancy [61], nor is there literature linking *HMCN1* to clinical outcomes. Therefore, this finding together with the association of differential methylation of *CRYGN* is highly novel and requires further validation.

We identified eight CD8^+^ T cell csDMPs that map to six genes and one intergenic region. The involvement of CD8^+^ T cells in MS pathophysiology is well established [59]. During pregnancy CD8^+^ T cells are critical for maternal–foetal tolerance and protection against viruses [63]. The functions and diseases associated with the CD8^+^ T cell csDMPs identified in this study suggest they are markers of pregnancy outcomes, rather than genes implicated in the modulation of MS outcomes due to pregnancy (e.g. *OR2L1, HOOK2 and CUL2)*. Most notably, *AHR* (cg25577322) is upregulated in decidual natural killer cells in women with recurrent spontaneous abortion and was hypomethylated in parous women in our study [64]. Here, we excluded pregnancies ending in miscarriage or termination to prevent identifying epigenetic biomarkers of miscarriage or termination. While it is possible that this signal was driven by unreported terminations and/or unknown miscarriages, it was identified in peripheral CD8^+^ T cells only (not whole blood) and is therefore unlikely to be a marker of recurrent spontaneous abortion in this cohort. Furthermore, multiple studies have recently correlated *AHR* agonist activity with MS subtype and prognosis [65, 66]. In these studies, *AHR* agonist activity increase was associated with relapse in CIS and RRMS [65]. A decrease in *AHR* agonist activity was associated with RRMS remission [65] and progressive MS [66], thus implicating *AHR* in neuroinflammatory processes. In our study, *AHR* was hypomethylated. Hypomethylation is often, but not always associated with upregulation of gene expression. Unfortunately, we did not assess gene expression. However, this provides a plausible mechanism by which pregnancy could modulate disease outcomes and warrants further investigation.

Ours is the first study to report a reduction in MAA in parous wwMS, compared to age-matched nulligravida wwMS. We demonstrated that parous women have a reduced mean MAA of between 1.44 to 2.27 years depending on the algorithm employed. This shows that, as in health, parity is associated with a reduction in MAA in wwMS [28]. GrimAge is the newest algorithm with robust associations with morbidity and mortality [34]. Furthermore, PhenoAge acceleration is associated with an increased risk of physical functioning problems [38]. Reduced MAA was not associated with years since conception in parous women, demonstrating that the benefit of parity on biological aging does not appear to weaken with time. Furthermore, MAA was not associated with menopause, suggesting that methylation changes associated with parity may not be reversed or otherwise impacted by the hormonal changes experienced throughout menopause. As a whole, our findings demonstrate slower biological aging in parous wwMS, and potentially a longer period of health and lifespan [38].

This is the largest study to date investigating the association between genome-wide methylation and parity in women with relapse-onset MS. We identified hundreds of methylation changes associated with parity that may underlie long-term outcomes in wwMS.

Cohort matching by age limited confounding and erroneous associations between methylation patterns and parity. We aimed to mitigate against confounding by disease severity by matching for ARMSS scores, therefore allowing us to study the relationship between methylation patterns and parity specifically. Whether these changes are specific to wwMS or a broader response to pregnancy remains to be confirmed in future studies including women without MS. We were underpowered to adjust for a range of clinical and environmental factors potentially associated with methylation patterns, including number of births and DMT [67], which could have contributed to residual confounding of our findings. Study power also limited our ability to identify small cell-type-specific effects using statistical deconvolution techniques, beyond those identified in T cells. Moreover, the use of algorithms that deconvolute immune cell subtypes at a more granular level (e.g. B naïve cells), such as IDOL-ext, may reveal more about the relationship between parity, cell-specific methylation patterns and clinical outcomes. Therefore, our findings require validation in a larger cohort of wwMS to address these limitations with adequate statistical power. As ours is a retrospective and cross-sectional study, we were not able to establish a causal link between pregnancy, methylation pattern changes and long-term clinical outcomes in wwMS. We are currently undertaking a longitudinal and prospective study of methylation changes during and after pregnancy relative to a nulligravida baseline, to investigate temporal and causal relationships between pregnancy, methylation and disease outcomes in wwMS. This could lead to the identification novel therapeutic targets.

## Conclusion

We investigated the association between whole blood and cell-type-specific genome-wide methylation patterns and parity in 192 women with relapse-onset MS. We identified small but potentially cumulative differences in whole-blood and T cell methylation patterns in genes related to neural plasticity, offering a putative molecular mechanism driving the long-term effect of pregnancy on MS outcomes. We further identified reduced methylation age acceleration in parous wwMS, demonstrating slower biological aging compared to nulligravida wwMS. As methylation patterns can be cell-type specific, our results suggest a potential ‘CNS signature’ of methylation in peripheral immune cells, as previously described in relation to MS progression [68]. This is the first genome-wide methylation study of parity in wwMS, and therefore, validation studies are needed to confirm our findings.

## Materials and methods

### Ethics approvals

Ethics approval for the collection of demographic, clinical, treatment and pregnancy history data via the MSBase Registry [31] was obtained from the Alfred Health Human Research Ethics Committee (528/12), and institutional review boards at all participating centres. Approval for the collection of genetic data was obtained from the Australian National Mutual Acceptance Scheme (HREC/13/MH/189). Written informed consent was obtained from participants as per local laws at each study site.

### Clinical data collection

This study utilised clinical data from the MSBase Registry, an international, prospective, observational MS clinical outcomes register. Data are collected in a unified manner and include patient demographics, Expanded Disability Status Scale (EDSS) scores, relapse, treatment and pregnancy data, as previously described [31, 32].

### Participant recruitment, parity definitions and sample collection

Whole-blood samples were obtained from 1984 participants. From this cohort, we selected 192 matched participants based on geographical location (Australia), sex (female), birth history availability (nulligravida or parous) and age (groups age-matched within three years, Additional file 1: Fig. S1).

DNA methylation is associated with chronological age [69], geographical location [69], immune cell-type proportions [70] and smoking [71]. To address this, we restricted participants to Australians matched by age (within three years). We further matched participants by disease severity as measured by Age-Related Multiple Sclerosis Severity (ARMSS) scores [72], due to non-negligible differences between sample groups (Table 1) and previously demonstrated associations between ARMSS score and methylation patterns [73]. Participants were matched using the *optmatch* package [74] in the R statistical environment. Smoking and cell-type proportions were adjusted for in the primary analysis, as described in below in section ‘Primary differential methylation analysis’.

The timing of pregnancy effects on methylation patterns remains unclear in wwMS, as does the impact of pregnancies resulting in miscarriage or termination compared to birth. We therefore excluded gravida women (i.e. those experiencing a miscarriage or induced abortion only) and restricted study inclusion to women who had at least one preterm or term birth prior to the date of blood collection, or those who were nulligravida. We included wwMS from the Royal Melbourne Hospital (VIC, *n* = 73), Box Hill Hospital (VIC, *n* = 56), John Hunter Hospital (NSW, *n* = 25) and Flinders Medical Centre (SA, *n* = 38). A total of 96 nulligravida and 96 parous females with RMS were included in this study (*n* = 192).

### DNA extraction

Each site extracted genomic DNA from whole blood using standard protocols and procedures.

### Methylation arrays

DNA samples were processed for methylation arrays at the Hunter Medical Research Institute (NSW). DNA quantity and quality were assessed using Qbit (Invitrogen™, USA) and TapeStation (Agilent™, USA), respectively. Samples meeting concentration and quality requirements were bisulphite converted using the EZ-DNA Methylation™ Kit (Zymo) according to manufacturer guidelines. Converted DNA was hybridised to Illumina Methylation EPIC BeadChip arrays (EPIC arrays). Samples were randomised based on clinic site using the *OSAT* R package to avoid batch effects. EPIC arrays were read using an iScan (Illumina™), and raw Idat files were produced for analysis.

### Genotyping arrays

Genomic DNA was sent from participating study sites to the Center for Genome Technology, John P. Hussman Institute for Human Genomics, University of Miami, for quality assessment and genotyping. Genotyping was performed in two batches using Illumina Multi-ethnic genotyping array (MEGA^EX^) arrays. Genotype calling was conducted in GenomeStudio v2.0 (Illumina).

### DNA methylation analysis pipeline

Our EWAS analysis was informed by the guidelines described in Campagna et al. (2021) [75]. The *Chip Analysis Methylation Pipeline (ChAMP)* Bioconductor package [76] was used for methylation data pre-processing in the R statistical environment. Raw Idat files were filtered to exclude low-quality samples (failed to successful probe ratio > 0.1), low-quality probes (negative detection *p* value > 0.01, bead count < 3 in ≥ 5% of samples), non-CpG probes, SNP-related probes, non-autosomal probes and multi-hit probes. Additional multi-hit probes were excluded based on Pidsley [77] (Additional file 2: Table S1). Beta values were normalised using the beta-mixture quantile (BMIQ) method [78]. Batch effects at the array and chip level were identified with singular value decomposition (SVD) analysis [79] and corrected for using the *Combat* algorithm [33].

### Primary differential methylation analysis

Differential methylation (Δ_meth_) between nulligravida and parous groups was identified at the single CpG level, i.e. differentially methylated positions (DMPs), and genomic region level, i.e. differentially methylated regions (DMRs), using the filtered and normalised beta matrix, as previously described [75]. We used a modified version of the *ChAMP* function *champ.DMP* to implement an adjusted logistic model of methylation level at each probe and parity group, adjusted for cell-type proportion estimates and DNAmPACKYRS due to the known confounding effect of these variables and significant differences between groups (Table 1) [71]. Cell-type proportions were estimated as described below in *Differential methylation analysis—immune cell specific*. DNAmPACKYRS is a biomarker of smoking, estimated based on methylation levels at smoking-associated CpGs. We used this biomarker due to incomplete self-reported smoking history data and calculated DNAmPACKYRS for each participant using the GrimAge online tool (https://dnamage.genetics.ucla.edu/home). A false discovery rate (FDR) threshold of 0.05 was used to assess statistical significance for all analyses. Methylation beta values equate to percentage methylation; therefore, we report methylation differences (effect size) as a percentage (e.g. Δ_meth_ of 0.01 = 1%). DMPs with an Δ_meth_ less than 1% were removed to avoid false positives produced from technical error.

We identified DMRs using a two-pronged approach. Firstly, with the *DMRcate* R package [80] using the following parameters: at least three DMPs within 1000 bp of the adjacent DMP, a DMP and DMR threshold of FDR < 0.05. Secondly, using the DMP list to identify at least five DMPs with an FDR < 0.01 and the same direction of effect, located within 1000 bp of each other. The validity of this strategy to identify DMRs in studies with small sample and/or effect sizes has previously been shown [35, 81].

### Differential methylation analysis—immune cell specific

We estimated cell-type proportions from whole blood methylation data using the *EpiDISH* R package [82] with the *CIBERSORT* algorithm [36] and Reinius (2012) reference dataset [83]. Cell-type proportion differences between nulligravida and parous groups were considered non-negligible if the Cohen’s *d* > 0.15.

Cell-type-specific DMPs (csDMPs) were identified using a modified version of the *cellDMC* function of *EpiDISH* [84]. *cellDMC* uses one linear model to identify csDMPs for all cell types, where the outcome is methylation level, and predictors are the proportion of each cell type and an interaction term of proportion and parity for the cell type of interest [84]*.* Below is an example for NK cells:

$$\begin{aligned}&csDMP_{NK} = M-value_{CpG} \sim Parity + NK\% + CD4T\% \\ &\quad+ CD8T\%+ B\% + Mono\% + Granulo\% + \left( {NK\% \times Parity} \right)\end{aligned}$$

To avoid overburdening the model due to our small sample size, we modified this by using one model per cell type. In each model, the outcome was methylation M-value, and the predictors were cell-type proportion estimate and an interaction term of cell-type proportion and parity. Below is an example for NK cells:

$$\begin{aligned}csDMP_{NK} &= M-value_{CpG} \sim Parity + NK\%\\&\quad + \left( {NK\% \times Parity} \right)\end{aligned}$$

Furthermore, we used M-values all cell-specific analysis, instead of beta values as per the whole blood analysis, as internal benchmarking showed a bias in cellDMC for identifying csDMPs in rarer cell subtypes which was attenuated with the use of M-values. A genome-wide threshold of *p* ≤ 9 × 10^−8^ was used to identify statistically significant csDMPs.

### Sensitivity analyses

Sensitivity analyses were performed to assess the potential impact of a series of demographic, clinical, biological and environmental covariates on the primary methylation analysis. Covariates were selected based on non-negligible differences between groups (Cohen’s *d* > 0.15), or a priori selected based on known associations with methylation patterns. Covariates included symptom duration, ARR and methylation age acceleration (PhenoAge and GrimAge). Environmental factors including treatment at blood collection (yes or no).

We calculated the difference methylation at each probe (Δ_meth_) between nulligravida and parous pairs matched by age and ARMSS score (96 pairs). The correlation between Δ_meth_ and covariate tested using Pearson’s correlation tests was used for continuous covariates and ANOVA tests for categorical covariates. For treatment at blood collection, pairs were required to have the same value for the correlation with methylation to be tested. Of 96 pairs in total, 40 pairs were on treatment at blood collection and 14 were off treatment. An FDR threshold of 0.05 was used to assess statistical significance for all sensitivity analyses.

### Single nucleotide variant analysis

Quality control was performed with *PLINKv1.9* [85]. Single nucleotide variants (SNVs) were excluded based on low call rate (< 95%), low minor allele frequency (MAF < 0.05), violation of Hardy–Weinberg equilibrium (*p* < 1 × 10^–5^), monomorphism and non-autosomal location. Samples were excluded based on sex inconsistencies, low call rate (< 95%) and relatedness (pi-hat > 0.05). Relatedness was assessed using identity by descent (IBD) analysis in *PLINKv1.9*, followed by confirmation in *KING* [86]. Principal components (PC) analysis was implemented in *EIGENSTRAT* [87]. PCs were projected to 1000 Genomes Project [88] data to assess population stratification effects and exclude population outliers.

### Methylation quantitative trait loci (mQTL) analysis

We extracted genotypes at SNVs located ± 5 kb up/downstream of DMR^Chr^ [17] and DMR^Chr^ [19] boundaries using the *KRIS* R package [89] and assessed linkage disequilibrium (LD) using bivariate correlations of genotype frequencies. The association between methylation and genotype at each DMR CpG-SNV pair was assessed using Kruskal–Wallis tests. We then performed general linear regressions with methylation as the dependent variable and genotype and parity as the independent variables to test if methylation was associated with parity independent of genotype at mQTLs. An p value threshold of 0.05 was used for all analyses.

### Multi-factor feature selection

We used machine learning to build an elastic net regression model to identify CpGs at which methylation was associated with parity, inputting beta values at 746,969 CpGs, cell-type proportions and DNAmPACKYRS. Samples were split into training (*n* = 134) and testing sets (*n* = 58) to reduce overfitting. The model was trained using a cross-validation resampling method with 10 iterations, with the train function of the *caret* R package [90]. The optimal alpha value was used in a subsequent k-fold cross-validation elastic net regression to identify the minimum lambda value; using the *cv.glmnet* function of the *glmnet* R package [91]. These alpha and lambda values were used in the final elastic net regression model that was applied to the testing set using the glmnet function of *glmnet* R package [91]. Features identified by the model to be associated with parity were compared to DMPs and DMRs identified in the primary analysis, as well as mapped to genes for GSEA performed as described below.

### Gene set enrichment analysis (GSEA)

We used gene set enrichment analysis (GSEA) to generate hypotheses about the functional consequence of differentially methylated genes between nulligravida and parous women. All CpGs that were associated with parity in the primary differential methylation analysis and elastic net regression were used as input. We conducted GSEA using two methods. Firstly, the *ToppGene* online application programming interface (API) [92] which takes an FDR ranked gene list ranked as input, with hypomethylated and hypermethylated genes analysed separately. Secondly, we used the *GOmeth* function [93] of the *missMethyl* R package [94] to address probe number and multi-gene bias specific to methylation data from arrays. A list of DMPs and all CpGs tested were used as input, and both Gene Ontology (GO) and KEGG pathway collections were tested. We used a Benjamini–Hochberg adjusted p value (FDR_B&H_) threshold of 0.05 to assess the statistical significance of enriched gene sets.

### Methylation age analysis

Methylation age is the prediction of biological age from methylation levels at a subset of CpGs (clock CpGs). PhenoAge [38] and GrimAge [34] are the most accurate and widely used methylation age algorithms and have been associated with increased risk of various morbidities and mortality [34, 37, 38].

We estimated methylation age using the PhenoAge [38] algorithm with the methyAge function of the *ENmix* R package [95]. GrimAge was calculated with the online calculator at https://dnamage.genetics.ucla.edu/. MAA was defined as the residual term from regressing chronological age on methylation age estimates. For each algorithm, Shapiro–Wilk normality tests were used to test the normality of the MAA distribution. To test if mean MAA was significantly different between groups, a one tailed t test was used for the PhenoAge algorithm, and a Mann–Whitney test for the GrimAge algorithm. We used a Pearson’s correlation test to test the relationship between years since conception and MAA in parous women. As menopause data were not available for this cohort, we divided women into under/over 50 years of age to study the association between menopause and MAA. A t test was used to assess statistical significance with a significance threshold of 0.05.

## Supplementary Information

**Additional file 1:** Supplementary Figures.

**Additional file 2:** Supplementary Tables.

## Data Availability

Supplemental files contain data supporting the conclusions in this article. Data access requests with scientifically sound proposals can be made in writing to Dr Vilija Jokubaitis (vilija.jokubaitis@monash.edu). Clinical data from the MSBase Registry: To protect participant confidentiality, de-identified patient-level data sharing may be possible in principle but will require permissions/consent from each contributing data controller.
