# Supplementary material for: Parity is associated with long-term differences in DNA methylation at genes related to neural plasticity in multiple sclerosis
Source: Clin Epigenetics. 2023 Feb 10;15:20. doi: 10.1186/s13148-023-01438-4 (PMC9921068; doi:10.1186/s13148-023-01438-4)
Supplement: Supplementary file 1 — Additional file 1: Supplementary Figures. [file 13148_2023_1438_MOESM1_ESM.pdf]

Supplementary Figures

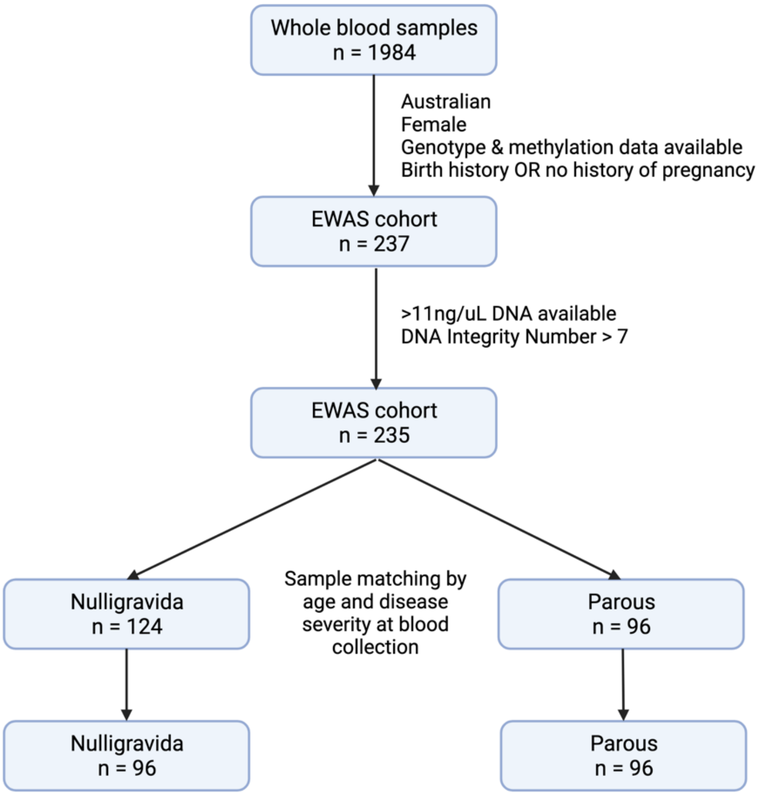

Supplementary Figure 1. Participant inclusion flowchart. Whole-blood samples were obtained from 1,984 participants. The cohort of 1,984 refined to 192 participants based on geographical location (Australia), sex (female), pregnancy history (nulligravida or parous), DNA quantity and quality, and age and disease severity at blood collection, as measured by Age Related Multiple Sclerosis Severity (ARMSS) scores. *Created with BioRender.com*

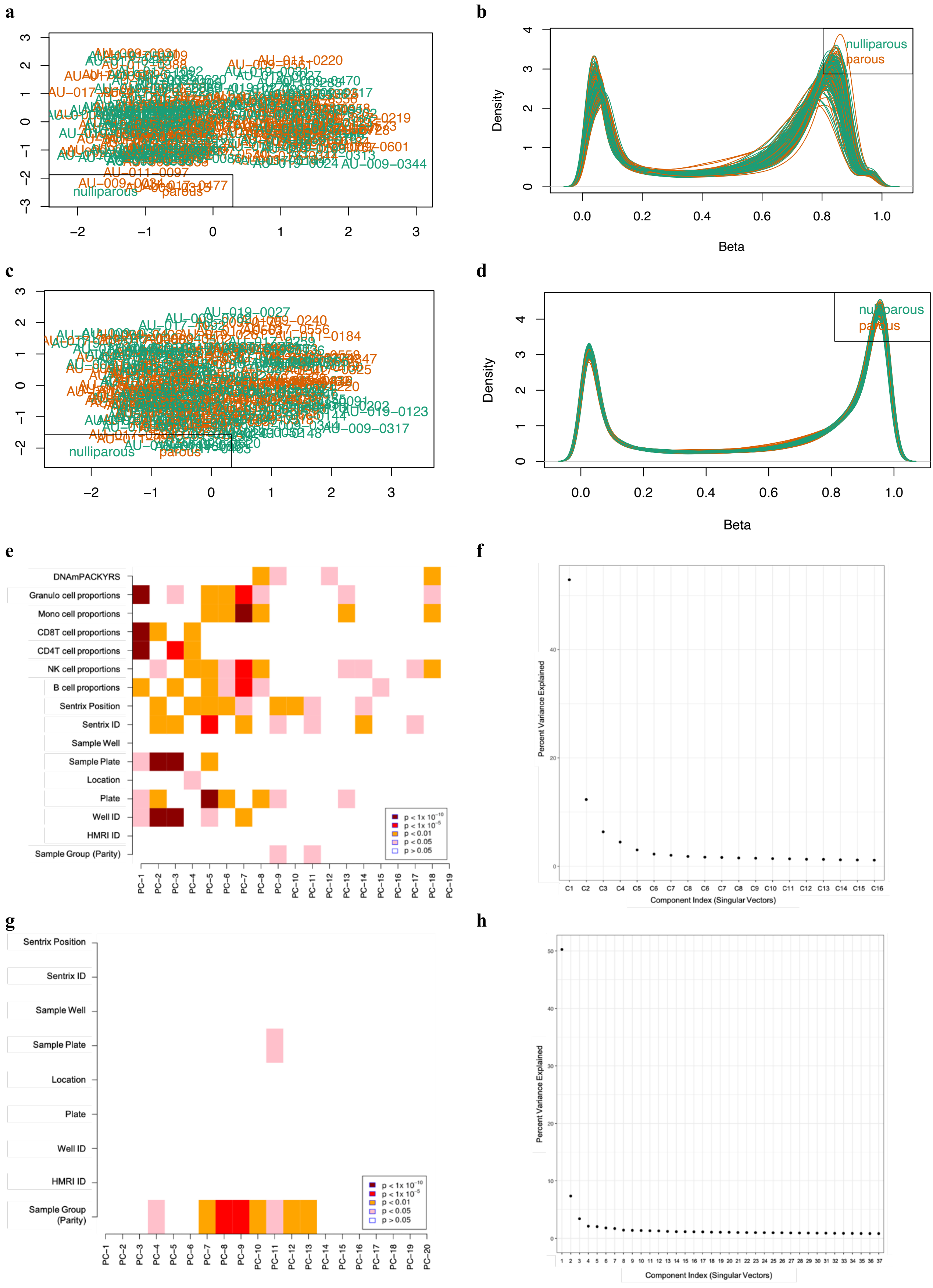

a
nulliparous
parous
b
Density
Beta
nulliparous
parous
c
nulliparous
parous
d
Density
Beta
nulliparous
parous
e
DNAmPACKYRS
Granulo cell proportions
Mono cell proportions
CD8T cell proportions
CD4T cell proportions
NK cell proportions
B cell proportions
Sentrix Position
Sentrix ID
Sample Well
Sample Plate
Location
Plate
Well ID
HMRI ID
Sample Group (Parity)
p < 1x 10^-10
p < 1x 10^-5
p < 0.01
p < 0.05
p > 0.05
PC-1 PC-2 PC-3 PC-4 PC-5 PC-6 PC-7 PC-8 PC-9 PC-10 PC-11 PC-12 PC-13 PC-14 PC-15 PC-16 PC-17 PC-18 PC-19
f
Percent Variance Explained
Component Index (Singular Vectors)
C1 C2 C3 C4 C5 C6 C7 C8 C6 C7 C8 C9 C10 C11 C12 C13 C14 C15 C16
g
Sentrix Position
Sentrix ID
Sample Well
Sample Plate
Location
Plate
Well ID
HMRI ID
Sample Group (Parity)
p < 1x 10^-10
p < 1x 10^-5
p < 0.01
p < 0.05
p > 0.05
PC-1 PC-2 PC-3 PC-4 PC-5 PC-6 PC-7 PC-8 PC-9 PC-10 PC-11 PC-12 PC-13 PC-14 PC-15 PC-16 PC-17 PC-18 PC-19 PC-20
h
Percent Variance Explained
Component Index (Singular Vectors)

Supplementary Figure 2. Quality control analysis of methylation data using ChAMP. a) Multidimensional Scaling (MDS) Plot of methylation beta values for all samples, with the 1000 most variable positions displayed. There is no grouping of nulligravida or parous groups evident **b)** A density plot of raw methylation data prior to normalisation, plotting the beta value distribution for all samples. **c)** MDS plot after beta value normalisation. **d)** Density plot after beta value normalisation. **e)** Singular Value Decomposition (SVD) heatmap using technical and biological variables to identify batch effets. **f)** Scree plot of components 1-16. **g)** SVD heatmap after batch effect correction for Plate, Sentrix ID and Sentrix Position using ComBat. After correction, Sample Group (parity) explained most of the variance in the data which was expected as the variable of interest. **h)** Scree plot of components 1-37 after batch effect correction.

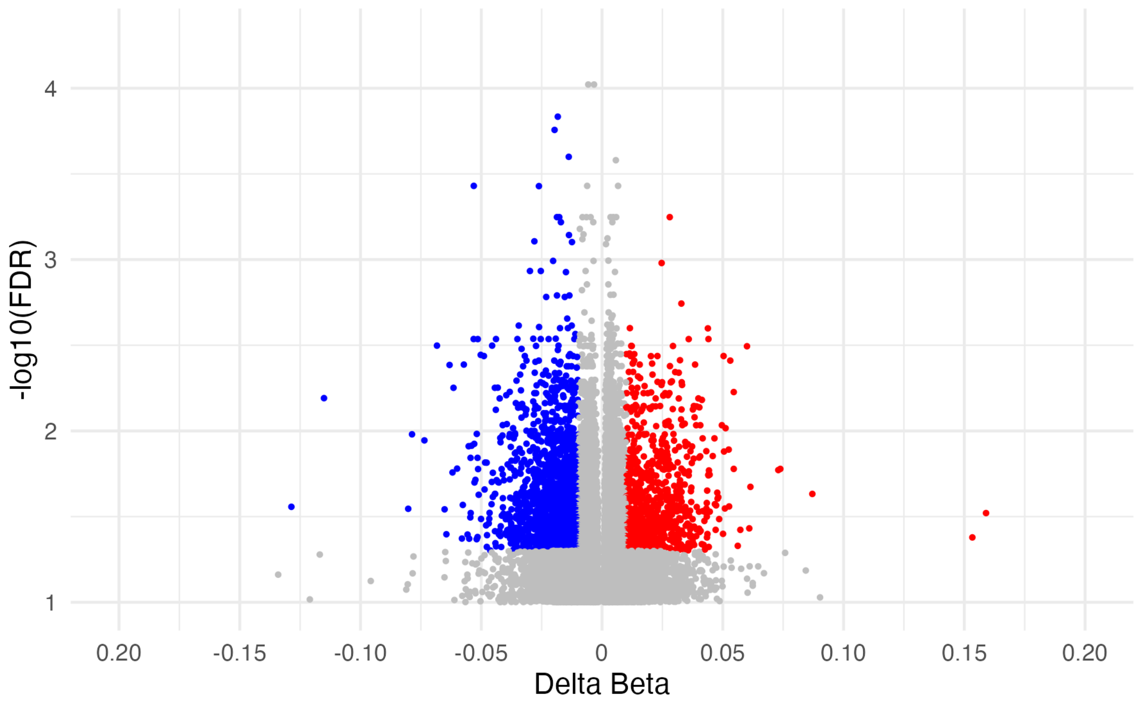

Supplementary Figure 3. Differentially methylated positions (DMPs) between nulligravida and parous groups. Of 2306 DMPs, 764 (33%) were hypomethylated (blue) and 1472 (67%) were hypermethylated (red) in the parous group. Delta beta (Δ_{meth}) ranged from –0.16 to 0.13. DMPs with Δ_{meth} above +/- 0.15 are labelled.

Abbreviations: FDR = false discovery rate

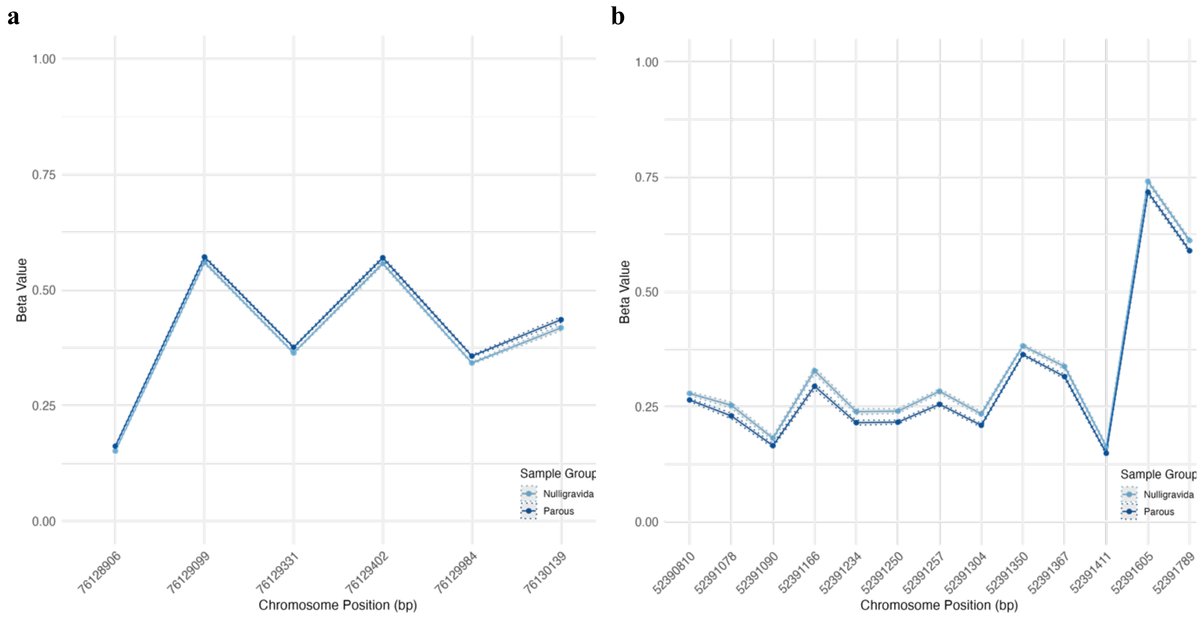

Supplementary Figure 4. Differentially methylated regions (DMRs) between nulligravida and parous groups. Mean methylation (β) values for nulligravida (light blue) and parous (dark blue) groups at each CpG in **a)** Chr17: 76128906-76130139, $\Delta_{max} = 0.017$, and **b)** DMR^{Chr15}: Chr19: 52390810-52391789, Δ_{max} = -0.014. Grey shading shows standard error of the mean.

a

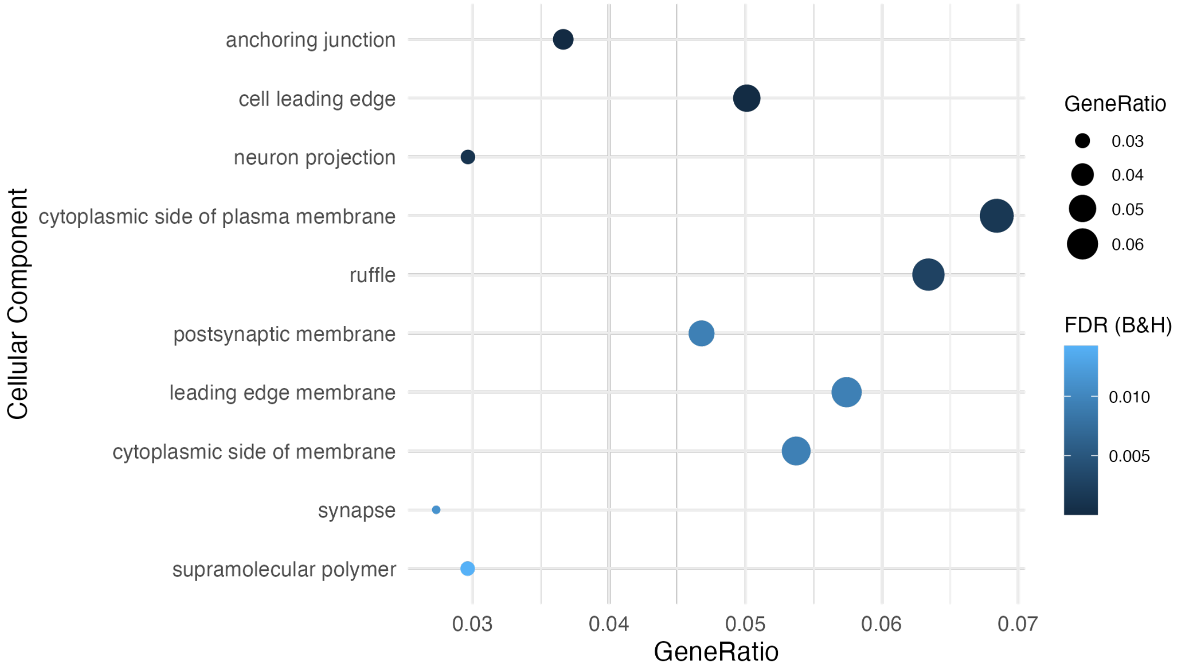

b

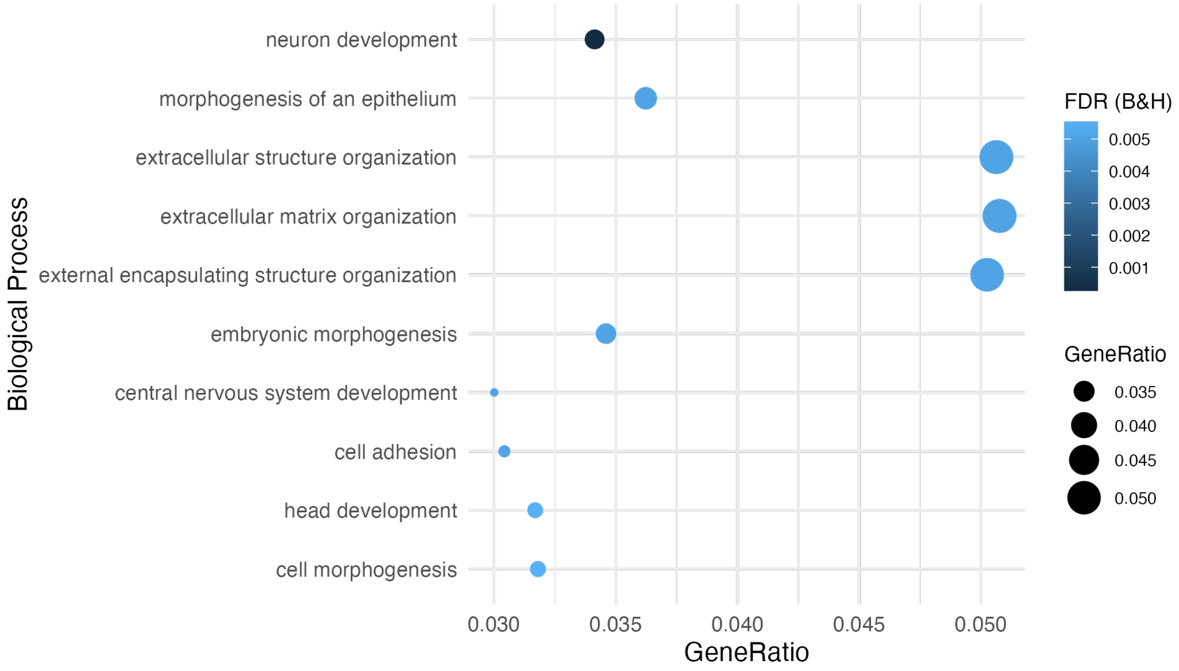

Supplementary Figure 5. Gene set enrichment analysis of validated genes from Mehta (2019, n=366). The **a)** ten most significantly enriched biological processes and, **b)** ten most significantly enriched cellular compartments. Gene ratio is the ratio of the number of genes in the query list and the hit count for that gene set in the genome.

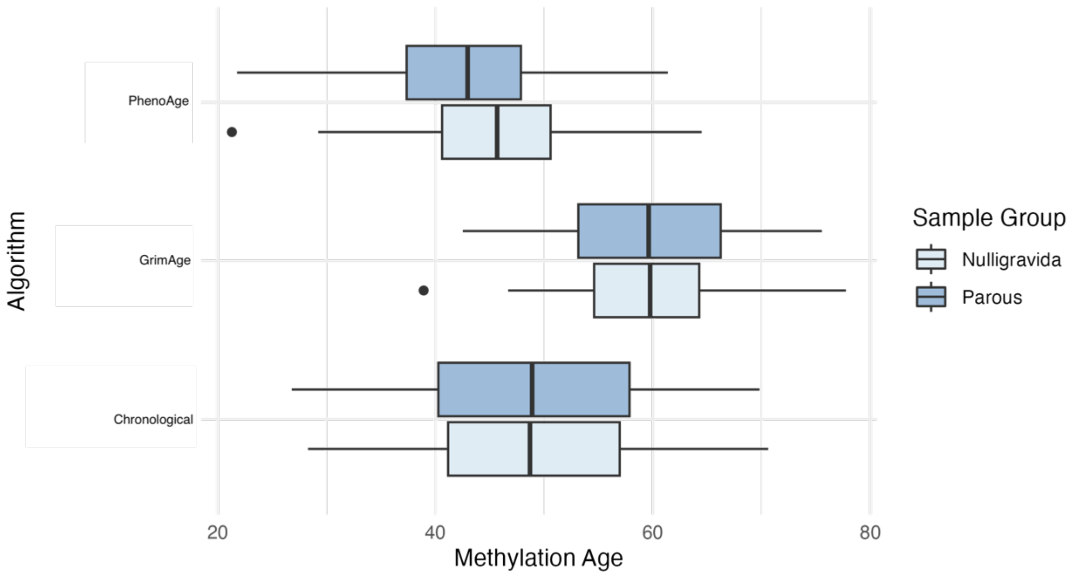

Supplementary Figure 6. Methylation age estimates and chronological age by sample group. Methylation age was estimated using the PhenoAge and GrimAge algorithms. Significant differences between groups were identified using the PhenoAge algorithm ($p = 0.034$). No significant differences between groups were identified using the GrimAge algorithm ($p = 0.854$).
